# Nightjars may adjust breeding phenology to compensate for mismatches between moths and moonlight

**DOI:** 10.1002/ece3.4077

**Published:** 2018-04-27

**Authors:** Philina A. English, Joseph J. Nocera, David J. Green

**Affiliations:** ^1^ Department of Biological Sciences Simon Fraser University Burnaby BC Canada; ^2^ Faculty of Forestry and Environmental Management University of New Brunswick Fredericton NB Canada

**Keywords:** adaptive phenology mismatch, aerial insectivore, *Antrostomus vociferus*, Caprimulgidae, double brood, whip‐poor‐will

## Abstract

Phenology match–mismatch usually refers to the extent of an organism's ability to match reproduction with peaks in food availability, but when mismatch occurs, it may indicate a response to another selective pressure. We assess the value of matching reproductive timing to multiple selective pressures for a migratory lunarphilic aerial insectivore bird, the whip‐poor‐will (*Antrostomus vociferus*). We hypothesize that a whip‐poor‐will's response to shifts in local phenology may be constrained by long annual migrations and a foraging mode that is dependent on both benign weather and the availability of moonlight. To test this, we monitored daily nest survival and overall reproductive success relative to food availability and moon phase in the northern part of whip‐poor‐will's breeding range. We found that moth abundance, and potentially temperature and moonlight, may all have a positive influence on daily chick survival rates and that the lowest chick survival rates for the period between hatching and fledging occurred when hatch was mismatched with both moths and moonlight. However, rather than breeding too late for peak moth abundance, the average first brood hatch date actually preceded the peak moth abundance and occurred during a period with slightly higher available moonlight than the period of peak food abundance. As a result, a low individual survival rate was partially compensated for by initiating more nesting attempts. This suggests that nightjars were able to adjust their breeding phenology in such a way that the costs of mismatch with food supply were at least partially balanced by a longer breeding season.

## 1 INTRODUCTION

Studies of reproductive phenology in seasonal environments often focus on an organism's ability to track food availability (Beaugrand, Brander, Alistair Lindley, Souissi, & Reid, 2003; Malick, Cox, Mueter, & Peterman, 2015; Pearce‐Higgins, Yalden, & Whittingham, 2005; Visser, van Noordwijk, Tinbergen, & Lessells, 1998). The match–mismatch hypothesis predicts that fitness will be highest when a consumer's reproductive demand peaks simultaneously with the availability of resources (Cushing, 1990). The strength of selection for a match between demand and availability will depend on the relative magnitude and duration of a seasonal peak in resource abundance (Durant et al., 2005; Vatka, Rytkönen, & Orell, 2014), but the outcome can be constrained by a species’ life‐history traits. For example, seasonal migrations could limit a species’ ability to advance reproduction, while particularly slow offspring development times could limit a species’ ability to delay reproduction (Both & Visser, 2001; Both et al., 2010). Furthermore, a mismatch between timing of reproduction and peaks in the availability of food resources could also reflect an adaptive response to some other selective pressure (Visser, te Marvelde, & Lof, 2012), like predators (Senner, Stager, & Sandercock, 2017; Toyama, Kotaka, & Koizumi, 2015), cold weather (Visser et al., 2015), day length (Varpe & Fiksen, 2010), or tidal flooding events (Lytle, 2002; Shriver, Vickery, Hodgman, & Gibbs, 2007).

Selective forces influencing reproductive phenology in seasonal environments are also likely to interact in complex ways. The accessibility of prey can be limited by weather or even day length (Varpe & Fiksen, 2010). Those individuals that can track food abundance will experience fitness benefits directly through improved offspring nutrition (Samplonius, Kappers, Brands, & Both, 2016) and indirectly through more efficient foraging that allows more time to defend their young (Duncan Rastogi, Zanette, & Clinchy, 2006; Zanette, Clinchy, & Smith, 2006). However, adjusting reproductive timing to match peaks in food abundance could also have fitness costs and therefore be maladaptive. For example, reduced recruitment could still result from successful tracking of an early peak in food abundance through increased risk of offspring mortality due to inclement weather (Winkler, Luo, & Rakhimberdiev, 2013), or from matching a later peak that limits time available for additional breeding attempts (Hoffmann, Postma, & Schaub, 2015) and juvenile growth (McKim‐Louder, Hoover, Benson, & Schelsky, 2013; Verboven & Visser, 1998). Alternatively, the risks associated with tracking early peaks in food abundance might be offset by the survival benefits of breeding when predators are less abundant (Senner et al., 2017), or when poikilothermic predators are less active (Toyama et al., 2015).

**Figure 1:**
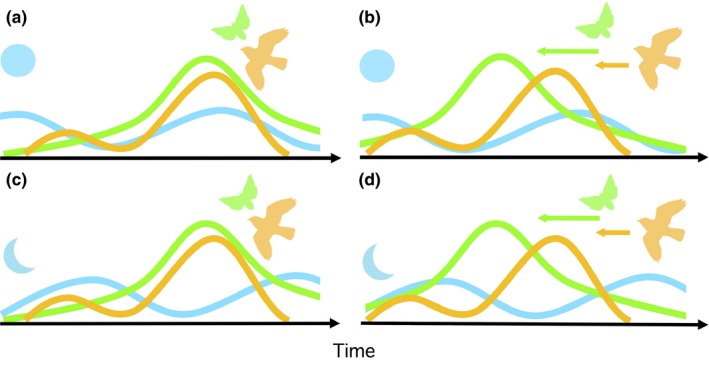
The hypothetical match–mismatch scenarios for an organism where fitness is influenced by multiple resources are illustrated for a lunarphilic nocturnal aerial insectivore: (a) complete synchrony between prey abundance (green), moon phase (blue), and predator demand (yellow) is expected to result in highest fitness for the predator, (b) asynchrony with prey abundance is partially compensated for by the synchrony between moon phase and predator demand, (c) mismatch with moon phase despite high synchrony between prey abundance and predator demand should reduce fitness, and (d) complete asynchrony between prey abundance, moon phase, prey, and predator demand should result in lowest fitness. Arrows indicate predicted direction and magnitude of temporal change in prey abundance and predator demand in response to a warming climate

Studies with long‐term datasets show high variability in the degree to which species’ phenologies, especially those depending on different trophic‐levels, can track changes in climate (Mayor et al., 2017; Thackeray et al., 2010, 2016). The result being population‐level mismatches between consumers and their resources, which can have demographic consequences for the species involved (Both et al., 2010; Hipfner, 2008; Plard et al., 2014). However, within most populations, there is some individual variation in the degree of matching between timing of reproduction and seasonal changes in the abundance of its resources, and whether matching is due to chance or choice, this variation can result in differences in fitness among individuals (Reed, Jenouvrier, & Visser, 2013; Reed et al., 2009). As individual fitness is influenced by multiple selective pressures, fitness should ultimately be highest for those individuals that can track multiple factors concurrently. For example, nocturnal aerially insectivorous birds might benefit from tracking temporal peaks in availability of food and the moonlight required for foraging (Figure 1).

**Figure 2:**
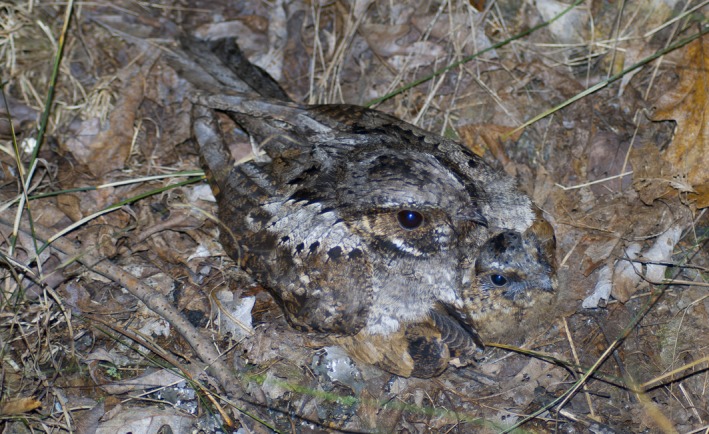
Female Eastern Whip‐poor‐will (*Antrostomus vociferus)* brooding two 15‐day‐old chicks

We assess the fitness consequences of matching reproductive timing to the availability of food availability and moonlight in the Eastern whip‐poor‐will (*Antrostomus vociferus,* hereafter “whip‐poor‐will”; Figure 2), a species of nightjar experiencing population declines in North America (Cadman, Sutherland, Beck, Lepage, & Couturier, 2007; Sauer et al., 2017). Nightjars are nocturnal aerially insectivorous birds that, unlike bats, are visual predators and must either rely on twilight periods, or moonlight for foraging (Jetz, Steffen, & Linsenmair, 2003; Mills, 1986). Some nightjars compensate for this highly restricted foraging period using torpor to reduce energy requirements when moonlight is absent (Smit, Boyles, Brigham, & McKechnie, 2011) and by timing reproductive energy demands for periods of the lunar cycle with the greatest moonlight availability (Jackson, 1985; Mills, 1986; Perrins & Crick, 1996; Vilella, 1995). Despite the synchronization between timing of reproduction and the availability of moonlight being expected to maximize fitness (Figure 1a,b), nightjars do not always time their reproduction to match the lunar cycle (Brigham & Barclay, 1992). This mismatch between reproductive phenology and moonlight could arise when other resources, like prey abundance (Figure 1c), or temporal constraints (Figure 1d) are more important. Still, it remains unknown how the extent to which a failure to match reproduction to availability of either resource influences the annual reproductive success of any nightjar species, or how they might respond by adjusting reproductive phenology when peaks in moonlight and food availability do not coincide (Figure 1b–d). We use estimates of daily nest (egg or chick) survival and annual productivity to assess the fitness consequences of matching reproduction to availability of both food and moonlight for a population of whip‐poor‐wills. We explore the degree to which this population tracks both resources by comparing overall availability of both food and moonlight with their availability after observed hatch dates. Finally, we calculate mean annual productivity across all nesting attempts per pair. We predict that whip‐poor‐wills will suffer fitness costs (lower daily nest survival and per pair productivity) if they cannot track both food and lunar cycle, but that an inability to track food availability will have greater fitness consequences than an inability to track the lunar cycle.

## 2 MATERIAL AND METHODS

### 2.1 Study species

Whip‐poor‐will diet consists of approximately 60% moths and 40% beetles usually captured on short flights from perches often on, or near, the ground (Cink, Pyle, & Patten, 2017; Garlapow, 2007), and aside from twilight periods around dusk and dawn, foraging activity is dependent on the availability of moonlight (Mills, 1986).

Male whip‐poor‐wills arrive on their more northern breeding grounds in late April or early May, with females arriving on average more than a week later (English, Mills et al., 2017). Once established, pairs tend to be stable and occupy a general purpose territory for the duration of a breeding season (Cink et al., 2017). First clutches are laid in late May or early June (Peck & James, 1983) and almost always contain two relatively conspicuous eggs laid directly on leaf litter, generally under the shade of trees or tall shrubs (Akresh & King, 2016). The adult's cryptic plumage provides excellent camouflage; consequently, nightjars rarely leave their eggs unattended except for brief foraging periods at dusk and dawn (Troscianko, Wilson‐Aggarwal, Stevens, & Spottiswoode, 2016). Males do not usually incubate, although they will visit the nest for brief periods (~5 min) at dusk and dawn (personal observation). Incubation lasts ~20 days (Akresh & King, 2016) and chicks begin perching and making short flights at roughly 16 days of age (Cink et al., 2017). Chicks can hop short distances when only a few days old and will often scatter in opposite directions when disturbed by a predator. This behavior, combined with aggressive distraction displays by the parent, often allows for partial survival of broods (personal observation).

### 2.2 Field site, nest searching, and monitoring

We estimated variation in timing and success of whip‐poor‐wills breeding in the northern part of their range by finding and monitoring nests over three years. Our study site was Queen's University Biological Station (QUBS; 44.467–44.567°N, 76.333–76.417°W) in eastern Ontario, Canada. QUBS encompasses >3,200 ha of deciduous forest and abandoned farmland in various stages of succession. Both habitats are punctuated by numerous small wetlands, lakes, and ridges topped with small rock barrens. These frequent forest gaps, combined with generally sparse understory vegetation, provide ideal whip‐poor‐will foraging habitat (English, Nocera, Pond, & Green, 2017).

The first male whip‐poor‐wills were detected at our study site on 28 April 2011, 18 April 2012, and 27 April 2013. During twilight and on moonlit nights, when adult males were actively foraging and singing, we used mist nets and song playback to capture whip‐poor‐wills (mostly males) and fitted them with a 0.6–0.8 g (<0.02% of body mass) NTQB‐3‐2 or Pip Ag376 radiofrequency transmitting tag (Lotek, Newmarket, ON, Canada). We attached the tags to the base of one of the central rectrices using waterproof glue and waxed dental floss at even intervals along the tag antenna (Wiktander, Olsson, & Nilsson, 2001). Tags were dropped naturally with tail feather molt at the end of the breeding season. Using radiotelemetry, we then mapped territories of individual males and searched those territories for nests using headlamps to spot eye‐shine. “Eye‐shine” is created by the tapetum lucidum membrane found in the eyes of many nocturnal vertebrates, which reflects a bright circle of light back toward an observer with a light source positioned near their own eyes. Nest searching began 20 May 2011 (first nest found 1 June), 15 May 2012 (first nest found 19 May), and 25 May 2013 (first nest found 2 June) and continued on all territories where early nests were found until there was no further evidence of new clutches being initiated. While nesting attempts that were depredated early in the incubation period could have been missed in all years, the average stage of incubation at which clutches were found was similar (2011: 6 days, 2012: 9 days, 2013: 8 days) suggesting that this source of error was consistent across years. New nests were found as late as 2 July 2011, 15 July 2012, 19 July 2013. Searching stopped earlier in 2011 because at that point all the females found on early nests were either roosting with their fledglings or already found to be renesting. We primarily searched for nests on dark nights (moon < 50% illuminated) starting at least 1.5 hr after sunset when parents were likely to have returned to the nest to incubate eggs or brood chicks, because chick eyes lack eye‐shine until after fledging. For nests found after hatch, we estimated chick age based on the extent or timing of pin feather growth. Due to variation in number of field assistants, we were able to thoroughly search 9, 15, and 9 territories, respectively, in each of the three years.

To monitor nest fates, we used motion‐activated 920 nm infrared cameras (Bushnell Trophy Camera model 119466) tied to a nearby tree, or to a metal stake when necessary, within clear view of the nest, but beyond the distance at which each individual whip‐poor‐will was readily flushed. These cameras used infrared beams that are outside the visible spectrum of birds and possible nest predators. Cameras were set to record 30 s of video when triggered on the high sensitivity setting with a 1 s retrigger interval. We also set the camera to automatically trigger every night at fixed 5‐min intervals during the first two hrs after sunset. During this period, the nest is usually left unattended for 30–50 min, providing footage of unattended eggs or chicks for determination of hatch dates. Even on the highest sensitivity setting, the camera was not triggered by most movements of the parents (probably because feathers provide too much insulation for body heat to be detected by the infrared sensor) or by predators like snakes. We could identify most mammalian predation events and all those that occurred during the preset recording intervals. Whip‐poor‐will chicks are semi‐precocial and sometimes moved outside the camera's view. Therefore, we checked cameras at 3‐ to 7‐day intervals, swapped out memory cards (and batteries when necessary), and repositioned the camera if the chicks had moved. These camera checks were also conducted on dark nights to allow the use of adult eye‐shine for locating chicks. Nightjars are very reluctant to flush (Troscianko et al., 2016), so we were usually able to setup and check cameras without flushing the parent. If eggs or chicks disappeared, but the camera did not capture a predation event, we searched the immediate nest area (~25 m radius) thoroughly on at least two occasions within 10 days of failure to confirm the loss, and after 1–2 weeks, we searched the whole territory for subsequent nesting attempts. Despite the mobility of chicks, the presence of a brooding parent with eye‐shine, during the dark periods of the night, makes it possible to find and monitor young chicks (Figure 2). Beyond 15 days of age, the reduced presence of an adult, along with an increasing ability to cross obstacles and move greater distances using flight, makes finding fledglings less reliable. Therefore, we only estimate chick survival from hatch up to 15 days of age and attempted to visit most nests on the 15th day of posthatch. We consider this the prefledging period. The number of chicks surviving this period was determined both for each nesting attempt and cumulatively across all attempts for each breeding pair. A few fledglings that were found only after reaching 15 day old were included in per pair productivity estimates despite not being included in estimates of daily survival rates.

All field data collection methods involving animals followed the safety protocols of the Ornithological Council (Gaunt et al., 1997) and were approved by the Simon Fraser University Animal Care Committee (protocol #1001B‐11).

### 2.3 Resource phenology

We sampled flying insects nightly from 1 May to 1 August of 2011 and 2012 at a single site at QUBS using malaise traps (standard size SLAM Trap II with bottom collectors from MegaView Science, Taiwan). This location was near a lake and wetland (<100 m) and immediately surrounded by forests and clearings (<30 m) of similar composition to the rest of the study area. Flight activity of nocturnal insects does not vary consistently with lunar cycle (Brown & Taylor, 1971; Hecker & Brigham, 1999; Schaefer, 1976), but could be predicted to be higher during brighter nights due to enhanced navigation potential (Warrant & Dacke, 2016) and reduced activity by bat species that are most vulnerable to visual predators (Appel, López‐Baucells, Magnusson, & Bobrowiec, 2017). However, traps that use light as an attractant tend to catch fewer insects around the full moon due to competition with background illumination (Yela & Holyoak, 1997). Therefore, we used passive malaise traps to avoid any bias from the use of attractants. We hung one trap each at heights of 2 m and 4 m to cover the most frequent foraging heights of whip‐poor‐wills (Garlapow, 2007). Each trap had a bottle at the top and bottom, which we attached at sunset and collected at dawn. The bottles were half filled with slightly soapy water to break the surface tension. The size of the bottle openings precluded the capture of larger insects (>4.5 cm), so we recorded only the total number of small‐ and medium‐sized moths and beetles captured each night. The size class distribution of whip‐poor‐will prey is currently undocumented, but boluses found in chick mouths included only small‐ and medium‐sized moths and no beetles (personal observation; Cink et al., 2017). Weather influenced some nightly captures, but we assume that any weather‐related reduction in insect captures would reflect the accessibility of insect prey to whip‐poor‐wills. We only included moth abundance in our subsequent analyses, because we captured far fewer beetles and found little seasonal variation in beetle abundance. Nightly moth captures in our traps were only weakly correlated with available moonlight (2011: *r*_p_ = −0.15, *N* = 89, *p* = .16; 2012: *r*_p_ = −0.26, *N* = 90, *p* = .01).

### 2.4 Available moonlight

Using the package *lunar* (Lazaridis, 2014) in R version 3.2.1 (R Development Core Team 2015), we calculated the relative amount of moonlight potentially available for each night of each breeding season at our study site. Available moonlight was estimated as the average percent of the moon face illuminated at one‐hour intervals above a threshold of 25% illuminated, based on observed activity thresholds (Brigham & Barclay, 1992; Jetz et al., 2003; Mills, 1986), multiplied by the number of intervals when the moon was at least 5° above the horizon. We applied this calculation to an average 6‐hr period of darkness occurring at our latitude during the breeding season. We do not have estimates of cloud cover for each night, because cloud cover has not been shown to significantly influence whip‐poor‐will activity (Mills, 1986) and some moonlight penetrates most cloud densities.

### 2.5 Weather variables

We used averages from the three nearest Environment Canada weather stations that shared a similar latitude (Centerville: 44.4, −76.91; Hartington IHD: 44.43, −76.69; Lyndhurst‐Shawmere: 44.52, −76.08) to estimate conditions at our study site. We downloaded daily measurements of temperature and precipitation from Environment Canada's Historical Climate Data website (http://climate.weather.gc.ca). Wind data were not collected at any of these stations, so we included an estimate of windiness based on the magnitude (over a threshold of 31 km/hr) of the maximum wind gust recorded at the Kingston station ~30 km away (44.22, −76.60). Any days missing from all three local stations were filled in with data from the Kingston station. We assessed the shared variation in four centered and scaled (by dividing each value by each variable's standard deviation) weather variables (mean minimum temperature, mean total precipitation, standard deviation in precipitation, and windiness) using a principal components analysis (PCA) implemented in the package *stats* in R. Both precipitation variables loaded positively on the first principal component (PC1) making it our measure of likelihood and amount of rain (Table 1). Minimum temperatures were negatively related to PC2 making it our measure of nighttime cool temperatures. Finally, strong winds were most positively associated with PC3. Cumulatively these first three components explained 92% of the variance in our weather measurements and were therefore included in our models of daily egg and chick survival.

**Table 1:** Loadings indicating the relative strength of correlations between the original variables and each principal component and the proportion of overall variance represented by each principal component

Weather variable	PC1	PC2	PC3	PC4
Total precipitation (mean)	0.89	0.07	−0.18	0.41
Total precipitation (standard deviation)	0.86	−0.15	−0.28	−0.39
Minimum temperature (mean)	0.19	−0.85	0.49	0.05
Maximum wind gust (>31 km/hr)	0.43	0.53	0.73	−0.08
Proportion of total variance	0.44	0.26	0.22	0.08

### 2.6 Daily survival analysis

We modeled daily survival rates (Dinsmore, White, & Knopf, 2002) using the Program MARK version 8.0 (White & Burnham, 1999) and calculated the cumulative expected survival, separately for a 20‐day incubation period and a 15‐day prefledging period over the 3 years of our study. Clutches were considered a single unit because >90% of failures were complete. To allow for partial predation and starvation, each chick was considered separately. While we recognize that the fate of two chicks from the same clutch is not completely independent, the effect of a sibling on survival is hard to predict. For example, the presence of a sibling could either increase the chance of survival if a predator was distracted while consuming a sibling, or decrease survival because feeding two chicks should require twice as many insects as feeding one. We used a year‐only model to calculate variation in expected survival between years and calculated confidence intervals on these estimates using the Delta method (Powell, 2007). To evaluate more complex candidate models, we used Akaike's information criterion corrected for small sample sizes (AICc; Hurvich & Tsai, 1989) and considered models to be well‐supported when they had a lower AICc score than the null model and were within 2 ΔAICc of the model with the lowest AICc score (Arnold, 2010). AIC is more appropriate than Bayesian information criterion when identifying useful parameters in the context of a complex ecological system where parameters are likely to have very small/tapering effects (Aho, Derryberry, & Peterson, 2014). None of the variables in these models were strongly correlated with each other within years (*r* < .31). Weather and moth abundance variables were standardized to allow estimation of the relative effects of 1 standard deviation in variation. Moonlight is retained as a score that represents each added hour of bright moonlight.

We use a hierarchical approach to our survival analysis (Dinsmore & Dinsmore, 2007). First, annual, seasonal, and age effects were evaluated using a set of time‐based daily survival models (6 for egg clutches and 12 for chicks). Candidate sets included a null model and all combinations of year, either a linear or quadratic effect of ordinal day of season, and chick age, where applicable. We excluded models with interactions due to their inability to estimate all parameters (Cooch & White, 2001). Next, we added independent and additive bivariate effects of each weather principal component and moonlight to the top time‐based model to form a candidate set (8 models each for egg clutches and chicks). We included only one weather variable in each model to limit overfitting of models. The top temporal model acted as the null model to control for unmeasured sources of annual or seasonal variation (e.g., changes in predator abundance or behavior). This makes the coefficients of additional variables more conservative, because we are only testing their influence within time periods with statistically similar survival rates.

Finally, for the two years for which we had estimates of moth abundance, our candidate set of daily egg and chick survival models included the top temporal model as a null model and all additive combinations of moth abundance and all variables included in well‐supported models from the earlier sets (2 models for egg clutches and 9 for chicks). We assessed whether annual and seasonal differences in survival could be explained by variation in food availability by comparing confidence intervals of temporal variables between models with and without different covariates. To assess the relative influence of all covariates on survival of eggs and chicks, we report confidence intervals of coefficients from the well‐supported models for each candidate set and summed model weights.

### 2.7 Interannual variation in phenology and productivity

For each year, we calculated the mean amount of moonlight available and mean moth abundance for 15‐day periods following all possible hatch dates for first broods (i.e., for every day between and including the earliest and latest observed first brood hatch dates across all years: 30 May to 29 June). Nests were considered to be first broods if they hatched within 15 days of the first nest hatched in a given year. This assumption was supported by having also found earlier nests for the pairs with all the 2011 and 2013 late nests, and 5 of 9 late nests in 2012. To assess phenology differences between years, we compared hatch dates for first broods, mean availability of moonlight and moths for all possible 15‐day prefledging periods, and mean availability of moonlight and moths for all actual observed 15‐day prefledging periods using analysis of variance (ANOVA) and post hoc Tukey's tests. For evidence of matching between breeding phenology and both resources within each year, we compared bootstrapped samples (10,000 subsamples of equal size to the number of nests found in a given year) against overall availability of moonlight and moth abundance for that year using Kolmogorov–Smirnov tests. This resulted in a distribution of *D* ^*+*^ statistics. We then calculated the same statistic for the actual observed hatch dates in that same year, and we report the proportion of random samples with equal or higher *D* ^*+*^ statistics as a *p*‐value. This method accounts for the unique distributions of resource availability in each year and the variation in the number of nests found between years. Finally, we report the mean availability of resources in each year against the per nest and per pair productivity for that year. We conducted all statistical tests in R version 3.2.1 (R Development Core Team 2015).

## 3 RESULTS

### 3.1 Do multiple resources influence daily nest survival?

We found a total of 38 whip‐poor‐will nests associated with 8–14 different pairs each year. Overall, cameras detected 8 disturbances: 5 at night and 3 in daytime. Eggs or chicks were lost to fishers (*Pekania pennanti*), raccoons (*Procyon lotor*), gray ratsnakes (*Pantherophis spiloides*), white‐tailed deer (*Odocoileus virginianus*), a porcupine (*Erethizon dorsatum*), and possibly ants. Whip‐poor‐wills were filmed successfully deterring two deer and one unknown predator, but failing to deter a fisher, another deer, and two snakes despite being present at the nest (see Videos [Link], [Link], [Link]).

For 26 nests found at the egg stage, all but four nests contained an initial clutch or brood size of 2. All single egg clutches were late season nests (2 in July 2012 and 2 in late June 2013). We documented 11 cases where the entire clutch disappeared: At least one clutch was eaten by a deer and three clutches were eaten by snakes. On one occasion, partial clutch loss occurred because an egg was either crushed or consumed by a passing porcupine. One egg failed to hatch in each of two clutches.

Models examining daily clutch survival over three years had an effective sample of 240 exposure days. Daily clutch survival did not appear to vary across years or within seasons (Table 2). The estimated daily clutch survival rate was 0.955 (95% CI 0.921–0.975) based on the null model, which for an incubation period of 20 days suggests a clutch survival rate of 40% (95% CI 18–62). Likewise, we found no effect of weather, moonlight, or moth abundance on daily clutch survival (Table 2).

**Table 2:** Daily nest survival model candidate sets for whip‐poor‐will egg clutches implemented in the program MARK

Model	*K*	AICc	ΔAICc	Weight	Likelihood	Deviance
Time‐based (3 years)						
{S(.)}	1	81.16	0.00	0.53	1.00	79.15
{S(day)}	2	83.10	1.94	0.20	0.38	79.05
{S(year)}	3	83.92	2.75	0.13	0.25	77.81
{S(day + day^2^)}	3	85.07	3.90	0.07	0.14	78.96
{S(year + day)}	4	85.98	4.82	0.05	0.09	77.81
{S(year + day + day^2^)}	5	87.98	6.82	0.02	0.03	77.72
+ weather and moonlight (3 years)						
{S(.)}	1	81.16	0.00	0.26	1.00	79.15
{S(rain)}	2	81.70	0.53	0.20	0.77	77.65
{S(temperature)}	2	81.99	0.83	0.17	0.66	77.94
{S(wind)}	2	83.08	1.92	0.10	0.38	79.03
{S(moon)}	2	83.19	2.03	0.09	0.36	79.14
{S(rain + moon)}	3	83.75	2.59	0.07	0.27	77.65
{S(temperature + moon)}	3	83.96	2.80	0.06	0.25	77.86
{S(wind + moon)}	3	85.12	3.96	0.04	0.14	79.02
+ food availability (2 years)						
{S(.)}	1	44.42	0.00	0.73	1.00	42.40
{S(moths)}	2	46.46	2.04	0.27	0.36	42.38

We monitored survival of 43 chicks (12 in 2011, 23 in 2012, 8 in 2013) from 24 nests. Twenty‐eight of these chicks, from 18 broods, survived to 15 days of age. We documented six cases of complete brood loss and five of partial brood loss (1 in 2011 and 4 in 2012) with a mean uncertainty of 2.8 days (range: 0–6) in the timing of death. Cameras only confirmed the death of chicks on two occasions: one chick eaten by a fisher and a pair of chicks eaten by a raccoon. One brood disappeared after appearing agitated by a swarm of ants and this pair initiated another nest approximately a week later. These three events occurred when chicks were between 4 and 8 days of age. Of those chicks with some fate uncertainty: One whole brood disappeared at 4–6 days (and the female laid a new clutch within a week), one pair lost 1 chick at 2–5 days and the other between 5 and 8 days, 5 other chicks (all from different broods, 4 with surviving siblings) disappeared between 6 and 10 days of age, and 3 chicks disappeared between 10 and 15 days. For these three oldest chicks, disappearance alone could indicate movement beyond the usual search radius. However, in two cases, observations of both parents roosting alone provided evidence that the chick was no longer alive. In the third case, no fate beyond the last observation was included in the daily survival models.

**Figure 3:**
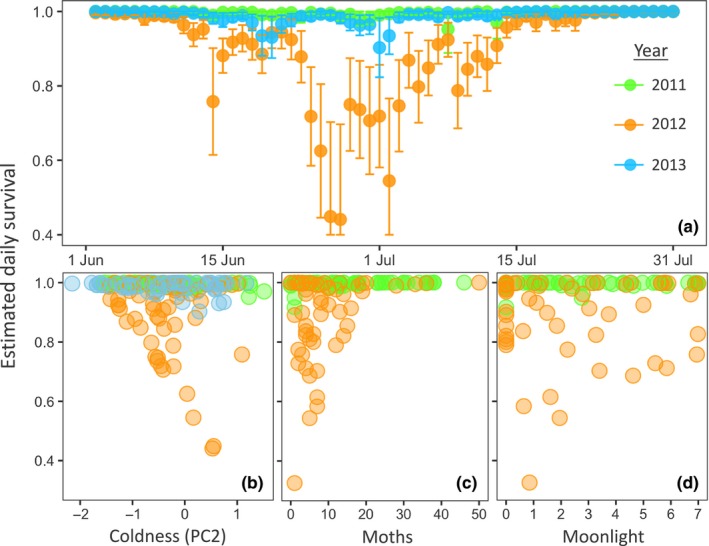
Daily chick survival estimates with 95% CI across the breeding season (a, 3‐year model) for the model controlling for the temperature weather variable (PC2; positive values indicate lower minimum temperatures, i.e., colder nights). The relationships between these daily survival estimates and either cooler temperatures (b, 3‐year model), moth captures (c, 2‐year model also controlling for moonlight and temporal effects), or moonlight (d, 2‐year model also controlling for moths and temporal effects)

Models of daily chick survival had an effective sample of 393 exposure days over three years. The estimated daily survival rate for chicks varied with year (model with year term has 12× the support of the null model based on model weights; Table 3) from a high of 0.993 (95% CI 0.955–0.999) in 2011 to a low of 0.940 (95% CI 0.893–0.968) in 2012 and was intermediate at 0.977 (95% CI 0.912–0.994) in 2013. Resulting estimated mean survival to 15 days of age varied from 91% (95% CI 73–100) in 2011 to only 40% (95% CI 17–63) in 2012, and 70% (95% CI 36–100) in 2013. Daily chick survival also varied seasonally, with survival being lowest in the middle of the breeding season and higher for both very early and very late nests (Table 3; Figure 3a). Daily chick survival models were improved by the addition of the temperature weather variable (PC2) alone (~2× the support of the best temporal model based on model weights; Table 3). Chick survival tended to be higher on nights with warmer temperatures (β = −1.27, 95% CI: −2.56 to 0.03; Figure 3b).

**Table 3:** Daily nest survival model candidate sets for whip‐poor‐will chicks implemented in the program MARK. Models with the temporal variable day^2^ include both day and day^2^ terms

Model	*K*	AICc	ΔAICc	Weight	Likelihood	Deviance
Time‐based (3 years)						
{S(year + day^2^)}	5	86.39	0.00	0.46	1.00	76.23
{S(year + day^2^ + age)}	6	87.56	1.17	0.26	0.56	75.34
{S(year)}	3	89.11	2.72	0.12	0.26	83.05
{S(year + age)}	4	90.81	4.42	0.05	0.11	82.71
{S(year + day)}	4	90.89	4.50	0.05	0.11	82.79
{S(year + day + age)}	5	92.56	6.17	0.02	0.05	82.41
{S(.)}	1	93.26	6.88	0.01	0.03	91.25
{S(day)}	2	94.46	8.08	0.01	0.02	90.43
{S(age)}	2	95.04	8.66	0.01	0.01	91.01
{S(day^2^)}	3	95.98	9.59	0.00	0.01	89.92
{S(day + age)}	3	96.34	9.95	0.00	0.01	90.28
{S(day^2^ + age)}	4	97.85	11.46	0.00	0.00	89.75
+ weather and moonlight (3 years)						
{S(year + day^2^ + temperature)}	6	85.16	0.00	0.34	1.00	72.94
{S(year + day^2^)}	5	86.39	1.23	0.18	0.54	76.23
{S(year + day^2^ + temperature + moon)}	7	87.06	1.90	0.13	0.39	72.77
{S(year + day^2^ + wind)}	6	87.57	2.41	0.10	0.30	75.35
{S(year + day^2^ + rain)}	6	88.08	2.92	0.08	0.23	75.86
{S(year + day^2^ + moon)}	6	88.14	2.98	0.08	0.23	75.92
{S(year + day^2^ + wind + moon)}	7	89.28	4.12	0.04	0.13	74.99
{S(year + day^2^ + rain + moon)}	7	89.77	4.61	0.03	0.10	75.48
{S(.)}	1	93.26	8.10	0.01	0.02	91.25
+ food availability (2 years)						
{S(year + day^2^ + moth)}	5	64.86	0.00	0.42	1.00	54.66
{S(year + day^2^ + moth + moon)}	6	66.22	1.36	0.21	0.51	53.95
{S(year + day^2^ + moth + temperature)}	6	66.53	1.67	0.18	0.43	54.25
{S(year + day^2^ + moth + moon + temp)}	7	68.05	3.20	0.09	0.20	53.68
{S(year + day^2^ + temperature)}	5	69.79	4.93	0.04	0.08	59.59
{S(year + day^2^)}	4	70.11	5.25	0.03	0.07	61.98
{S(year + day^2^ + moon + temperature)}	6	71.60	6.74	0.01	0.03	59.32
{S(year + day^2^ + moon)}	5	71.99	7.13	0.01	0.03	61.79
{S(.)}	1	77.53	12.67	0.00	0.00	75.52

For the two years with daily food availability estimates, the model including only moth abundance had 10× the support (based on model weights) of the model with the temperature weather variable (Table 3). Models including moth abundance and moonlight, or moth abundance and temperature, received similar support at about half that of moths alone (Table 3). Moth abundance was consistently estimated to have a positive effect (β for moth controlling for temporal variables = 1.72, 95% CI −0.05 to 3.49; Table 4), and the estimate of this effect no longer overlapped zero when moonlight was included in the model (β for moth controlling for moonlight and temporal variables = 2.03, 95% CI 0.002–4.05; Figure 3c; Table 4). Summed model weights (*w*) also provide some evidence that all three parameters could influence chick survival: moths (∑*w*_*i*_ = 0.91), moonlight (∑*w*_*i*_ = 0.31), and temperature (∑*w*_*i*_ = 0.32; Table 3). The effective sample size of the two‐year models was 309 exposure days.

**Table 4:** Coefficient estimates ß with (95% confidence intervals) for all daily nest survival models for whip‐poor‐will chicks in the two years with data on moth abundances. All models reported have greater support than the temporal null model (Δ 2AICc = 5.25) and contain both year and a quadratic effect of day of season (see Table 3 for the equations describing these models). Positive PC2 values indicate lower minimum temperatures**,** that is, colder nights

Model	Year	Day	Day^2^	Moths	Moon	PC2
Moth	−2.65 (−5.22 to −0.58)	−0.44 (−0.81 to −0.07)	0.0086 (0.0015–0.016)	1.72 (−0.04 to 3.49)		
Moth + moon	−2.97 (−5.41 to −0.54)	−0.491 (−0.910 to −0.072)	0.0094 (0.0015–0.017)	2.03 (0.00–4.05)	0.18 (−0.26 to 0.61)	
Moth + PC2	−2.86 (−5.16 to −0.56)	−0.489 (−0.897 to −0.081)	0.0091 (−0.0008 to 0.017)	1.61 (−0.19 to 3.41)		−0.55 (−2.20 to 1.09)
Moth + moon + PC2	−2.87 (−5.28 to −0.46)	−0.523 (−0.970 to −0.076)	0.0098 (0.0016–0.018)	1.92 (−0.13 to 3.98)	0.17 (−0.29 to 0.62)	−0.43 (−2.05 to 1.19)
PC2	−3.47 (−5.81 to −1.12)	−0.42 (−0.80 to −0.049)	0.0074 (0.0008–0.014)			−1.23 (−2.67 to 0.21)
Temporal null	−3.07 (−5.28 to −0.86)	−0.28 (−0.55 to 0.003)	0.0051 (−0.0001 to 0.010)			

### 3.2 Is interannual variation in phenology and productivity related to food availability and moonlight?

**Figure 4:**
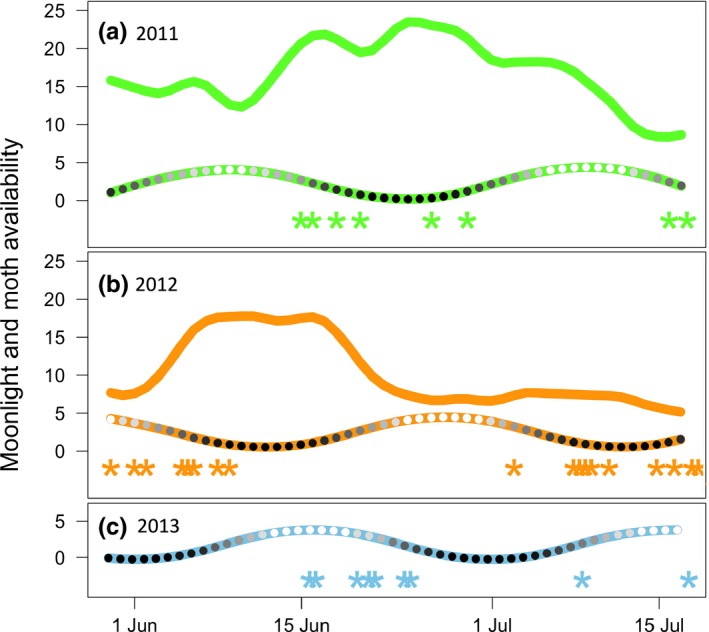
Annual differences in food availability (solid line: nightly moth counts averaged across each 15‐day prefledging period) and moon phase (beaded line: height of the line indicates hours of moonlight and lighter shaded circles indicate greater proportion of moon face illuminated for each 15‐day prefledging period) relative to observed hatch dates of whip‐poor‐will nests (star symbols) for each year (a, 2011; b, 2012; c, 2013). Nests clustering earlier in each season were considered first attempts in analyses

We were able to estimate hatch dates for six‐first brood nests in 2011, eight nests in 2012, and seven in 2013 (Table 5; Figure 3). Nests in 2012 hatched an average of 15 days earlier than in the other two years (*F*_2,18_ = 32.9; *p* < .001), and hatch dates were 8 and 23 days earlier in 2012 than in 2013 for the only two males for which early nests were found in both years. Mean moth abundance was higher in 2011 than in 2012 for both possible (Table 5; *F*_1,60_ = 33.7; *p* < .001) and for actual hatch dates (*F*_1,12_ = 24.5; *p* < .001). Whip‐poor‐wills matched hatching to peak food availability in 2011, but not 2012 (Figure 4). Moth abundance was significantly higher than could be expected by chance during actual whip‐poor‐will prefledging periods only in 2011 (2011: *D* = 0.52, *p* = .04; 2012: *D* = 0.17, *p* = .88). Whip‐poor‐wills appear to match hatching to moon phase in only one of three years. The overall availability of moonlight during the breeding season did not vary significantly between years (*F*_2,90_ = 0.11; *p* = .90), but the amount of moonlight available during prefledging periods of individual whip‐poor‐will nests did differ between years (*F*_2,18_ = 3.8; *p* = .041) and was significantly higher than could be expected by chance only in 2013 (2011: *D* = 0.39, *p* = .22; 2012: *D* = 0.19, *p* = .81; 2013: *D* = 0.484, *p* = .04).

**Table 5:** Annual difference in breeding phenology, productivity, and resource availability for whip‐poor‐wills breeding in southeastern Ontario, Canada (variance is reported as ± standard deviation, *N* = the sample size of nests or pairs for which estimates were possible, * = significance with superscripts denoting where differences are found). Moonlight score is a product of moon face illumination and hours spent above the horizon. Moth abundance was estimated from nightly aerial malaise trap captures

Year	2011	2012	2013
Breeding phenology and productivity estimates			
Mean (and range) for first brood hatch dates***	21 June^A^ (15 June–29 June) *N* = 6	4 June^B^ (30 May–9 June) *N* = 8	20 June^A^ (16 June–24 June) *N* = 7
Fledglings produced per nest	1.10 ± 0.88 *N* = 10	0.61 ± 0.78 *N* = 18	0.60 ± 0.97 *N* = 10
Fledglings produced per pair	1.56 ± 0.88 (0–3) *N* = 9	1.22 ± 0.97 (0–3) *N* = 9	1.33 ± 1.03 (0–2) *N* = 6
Proportion of pairs with multiple breeding attempts	0.33 *N* = 6	0.67 *N* = 9	0.33 *N* = 6
Proportion attempted 2nd brood after successful 1st brood	0.20 *N* = 5	0.57 *N* = 7	0.50 *N* = 2
Prefledging period resource availability (June–July)			
Mean moonlight score per night for all possible prefledging periods	2.20 ± 1.40	2.21 ± 1.45	2.06 ± 1.45
Mean moonlight score per night during actual prefledging periods*	1.63 ± 0.92^A^	2.13 ± 1.23^A^	3.18 ± 0.88^B^
Mean moth abundance for all possible prefledging periods*	18.35 ± 3.81	12.11 ± 4.61	–
Mean moth abundance during actual prefledging periods*	21.53 ± 1.18	12.54 ± 4.32	–

We were able to find first brood nests and to monitor territories for subsequent nesting attempts for a subset of pairs (Table 5); between 33% (2011 and 2013) and 67% (2012) of these pairs made more than one nesting attempt per year, and between 20% (2011) and 57% (2012) of pairs that fledged a first brood attempted a second brood. The average number of fledglings produced per nest (first and second broods combined) was highest in 2011 at 1.10 of a maximum of 2 and lowest in 2013 at only 0.60 (Table 4; *F*_2,35_ = 1.23; *p* = .31). As a result of multiple nesting attempts, per pair productivity ranged from 0 to 3 and the mean differed less across years, from 1.56 in 2011 to 1.22 in 2012 (Table 5).

## 4 DISCUSSION

Most studies of phenological match–mismatch only consider the need to match reproductive demand to the availability of one resource (e.g., Dunn, Winkler, Whittingham, Hannon, & Robertson, 2010), but for most species, the selective pressures on the timing of breeding are likely far more complex (Visser et al., 2012). Aerial insectivore birds in temperate climates may be particularly sensitive to the consequences of global climate change, including changes in seasonal phenology, because they must make long‐distance seasonal migrations and are dependent on insect prey that is only accessible when weather conditions allow for flight (Nebel, Mills, McCracken, & Taylor, 2010). Our study illustrates how multiple factors are likely to influence breeding phenology for nocturnal aerial insectivores, like whip‐poor‐wills, which face the additional challenge of only being able to forage during twilight periods, or when adequate moonlight is available. We show that while fitness benefits are likely to result from matching reproduction to temporal variation in food availability, and possibly moonlight, whip‐poor‐wills only appear to match reproduction to food availability and/or moonlight in some years. This evidence, that timing of breeding and variation in nest survival rates of whip‐poor‐wills could be linked to temporal availability of multiple resources across only a few years, highlights the need to consider multiple resources and selective pressures when attempting to understand how individuals and populations respond to changes in seasonal phenology.

This study provides some support for the hypothesis that nocturnal aerial insectivore populations could be sensitive to mismatches between timing of reproduction and seasonal changes in food availability. Daily survival rates of whip‐poor‐will chicks were positively related to prey abundance and averaged higher in 2011 when mean moth abundance was highest. Whip‐poor‐will reproductive phenology was only matched to a seasonal peak in food availability (Figure 1c) in this year. As expected, estimates of both mean daily survival and productivity were lower when reproduction and food availability were mismatched in 2012 (Figure 1d), however this year also had lower mean insect abundance. Furthermore, the negative effect of cooler temperatures on nestling survival could also be related to reduced insect activity. This is supported by a smaller parameter estimate when moths are included in the model (Table 4). These patterns are consistent with the wide variety of bird species that have been shown to have lower annual productivity when breeding is mismatched with peaks in prey abundance (McKinnon, Picotin, Bolduc, Juillet, & Bêty, 2012; Pearce‐Higgins et al., 2005; Reed et al., 2009, 2013; Verboven, Tinbergen, & Verhulst, 2001). Such failure to track prey availability could also lead to slower chick growth rates (Hipfner, 2008; McKinnon et al., 2012) and lower recruitment (Reed et al., 2013).

The fitness benefits of matching reproduction to seasonal peaks in food availability could be a direct result of an increase in the quantity of food provided to chicks. Another possibility is that increased fitness when food is more abundant could be an indirect result of more efficient foraging allowing more time for parents to defend the nest (Duncan Rastogi et al., 2006; Zanette et al., 2006). For whip‐poor‐wills, both mechanisms appear likely because one parent often broods chicks when not foraging, and chicks are frequently left unattended during peak foraging periods (personal observation). While we found no dead chicks, some of the unexplained brood losses (4 complete and 5 partial) could have been due to starvation or poor nutrition. In contrast, a range of predators were confirmed as proximate causes of nest failure. Therefore, we suspect that much of the positive influence of moth abundance on daily survival is due to parents spending less time away from the nest on nights when higher moth activity increases foraging efficiency. While defense against some predators, like a fisher or raccoon, may be somewhat futile, we observed an aggressive distraction display successfully directed at a deer. We suspect that this level of defense could allow at least one chick to escape detection by a variety of predators. Differences in chick survival between years and lowest survival in the middle of the season could also be related to local differences in predator activity and availability of alternative prey (Camacho, Sáez‐Gómez, Potti, & Fedriani, 2017). For example, turtle nests (possibly *Chelydra serpentine*,* Chrysemys picta*, and *Graptemys geographica*) are abundant at our study site (personal observation), which could attract predators like raccoons. To the extent that the lack of an effect of either weather or moths on egg survival is not just due to a lack of statistical power, it also supports a greater role for parental attendance when the contents of the nest are better camouflaged and able to flee (chicks vs. eggs). A role for parental defense is also consistent with most studies having found that the majority of nightjar nest failures occur during the egg stage (Cuadrado & Domínguez, 1996; Langston, Liley, Murison, Woodfield, & Clarke, 2007; Vilella, 1995) and 3 cases of partial brood loss versus no complete brood losses being found in the only study that reports individual chick survival rates (Langston et al., 2007).

If foraging efficiency is influencing fitness, we could expect that nightjars would benefit from matching reproduction to both food availability and lunar cycle (Figure 1a). Evidence that reproduction is timed to maximize available moonlight following hatching has been found for at least one temperate (Cresswell, 1992; Perrins & Crick, 1996) and a few tropical and subtropical nightjar species (Jackson, 1985; Pople, 2014; Vilella, 1995). However, the combined influence of moonlight and food availability has only been explored for Standard‐winged Nightjars (*Macrodipteryx longipennis*), a tropical lekking species with no paternal care. Within a period of peak food availability that spanned two lunar cycles, hatching was more likely to occur around the new moon, about one week earlier within a lunar cycle than found for whip‐poor‐will and most other nightjar species. This was posited to either reduce the risk to young hatchlings from visual predators or to maximize availability of moonlight for provisioning of two‐week‐old chicks (Jetz et al., 2003). When only one parent is provisioning, moonlight would only influence total provisioning potential and therefore would be more critical when chicks are larger and require more food (Goodbred & Holmes, 1996). Also, as tropical regions experience much shorter twilight periods (Mills, 2008), moonlight should be especially critical during periods of maximum nutritional demand (Jetz et al., 2003). Alternatively, a similar pattern could result if optimal conditions for assessing male quality influences timing of breeding (Penteriani, del Mar Delgado, Campioni, & Lourenço, 2010; Pople, 2014). Most other nightjars, including whip‐poor‐will, have biparental care such that more hours of moonlight would allow parents to alternate foraging and nest defense with each other, rather than both having to forage exclusively at dawn and dusk. For temperate breeding whip‐poor‐wills, the hours and brightness of moonlight appeared to have some potential to improve daily nest survival rates, but only when controlling for nightly moth abundance. The weakness of this effect may be due to a greater fitness benefit from matching reproduction with food availability than to moonlight alone, and the peaks in availability of moths and moonlight being out of sync with each other in both years for which we had data on moth abundances (Figure 3). Consistent with this explanation, the timing of reproduction was not matched to moonlight in either of these years. In 2013, timing of hatch did allow more hours of moonlight for provisioning of chicks during the prefledging period than would be expected by chance. That this synchronization between hatching and moonlight occurred in only one of three years suggests that while timing breeding to maximize available moonlight could benefit temperate breeding nightjars (as suggested by Mills, 1986), there seem to be other more important factors determining timing of breeding.

For long‐distance migrant birds, the match–mismatch hypothesis predicts that timing of arrival on the breeding grounds can constrain their ability to track seasonal advances in food availability, which can lead to a mismatch that reduces reproductive success (Both & Visser, 2001; Both et al., 2010; Møller, Rubolini, & Lehikoinen, 2008). Therefore, we would expect mismatches to be more likely in years where migrants arrive on the breeding grounds late. Surprisingly, the reverse appears true for whip‐poor‐wills, where a mismatch between reproduction and food availability occurred in 2012, when birds arrived relatively early and some chicks hatched more than two weeks ahead of the peak in food availability. This suggests that additional selective pressures must favor early breeding (Cooper, Murphy, Redmond, & Dolan, 2011). For whip‐poor‐wills, these factors could be higher levels of moonlight available to the earliest nests in 2012 (Figure 4b) and the opportunity to attempt multiple broods. After a nest failure, or successfully rearing a first brood, whip‐poor‐wills often lay a second, or even third clutch of eggs. As such, an individual's reproductive success per season is determined both by the number of offspring produced per nesting attempt and the number of breeding attempts (Nagy & Holmes, 2004). Although young hatched from second broods may have lower chances of surviving to reproduce themselves, partly because they have less time to mature before autumn (Møller, 1994). In 2012, possibly facilitated by earlier arrival on the breeding grounds (Cooper et al., 2011; Drake, Rock, Quinlan, & Green, 2013; Rockwell, Bocetti, & Marra, 2012), hatch dates were about two weeks earlier than in both the other years, which appeared to allow extra time for attempting to renest and raise multiple broods. The result being that, despite 51% lower nest survival rates, the per pair productivity was only 22% lower in 2012 than in 2011. However, there can also be costs to early breeding (Visser et al., 2015). Due to repeated periods of cold and rain in early and mid‐June of 2013, 90% of nests of Tree Swallows (*Tachycineta bicolor*), another aerial insectivore species breeding at the same location, failed due to starvation, hypothermia, and/or abandonment (Ouyang et al., 2015). Later hatch dates for whip‐poor‐wills meant that during this same time period most nests had not yet hatched. Thus, later breeding in 2013 could represent a successful trade‐off between the increased risks of harsh weather and the increased opportunities for double brooding that were seen in 2012 (Verboven et al., 2001). Nonetheless, numerous studies have found that individuals that arrive early have higher reproductive success (McKellar, Marra, & Ratcliffe, 2013) and that chicks hatched earlier are more likely to survive (Bowers et al., 2016; Naef‐Daenzer, Widmer, & Nuber, 2001; Verboven & Visser, 1998; Verhulst & Tinbergen, 1991).

We sought to present the hypothesis that multiple fluctuating variables can influence the consequences of phenology mismatch and we provide an example of how these interactions can be assessed. However, our study has limitations. First, an accurate assessment of nocturnal food availability for aerial insectivores is made challenging by the need to sample flying insects only at night and to avoid the use of attractants, like light, that can be biased by covariates of interest, like moonlight. Uncertainty regarding the precise diet of whip‐poor‐wills adds to this challenge. Malaise traps set and checked nightly overcame these hurdles, but are size‐limited to catching small‐ and medium‐sized insects. These size classes appear to dominate the food provided to chicks (Cink et al., 2017), but likely exclude some of the insects consumed by adults. Furthermore, our sampling was limited to three years at one study area. Future studies would benefit from improved trap designs, and even automation, that could allow sampling a greater diversity of insect size classes at multiple locations and over a longer period. Secondly, due to relatively large territory sizes (3–11 hectares; Cink et al., 2017), our sample size of nests is small and, as a result, the confidence intervals on the effects of variables included in well‐supported models remain large. Still, the occurrence of failure events throughout the season (with no two broods failing on the same night) on multiple days with low levels of food abundance still allows detection of relevant trends. Finally, we treat the fates of individual chicks from the same brood as independent, although they are exposed to more similar conditions and threats than unrelated chicks. We feel this is justified due to more cases where only one chick in a two‐chick brood survives than where both chicks die (5 vs. 3 of 19 two‐chick broods), and because this is the only modeling approach that allows us to detect partial predation and starvation events using established nest survival modeling techniques. Despite these limitations, we found support for models of nestling survival that include temporal effects, food abundance, and moonlight suggesting that discussions of these effects are warranted. Future work should test this approach in other study locations, a greater number of years, and in other lunarphilic species. Furthermore, detailed study of chick growth rates and flight ability would allow more direct assessment of the mechanisms and fitness consequences of mismatching breeding phenology, food abundance, and moon phase.

Cumulatively, the results of this study suggest that the complex pressures associated with lunarphilia and exploiting an environmentally sensitive food supply may have helped prepare migratory nightjars for a changing climate by selecting for flexibility in their timing of breeding (Camacho, 2013). For whip‐poor‐wills, across only three years, we find considerable variation in daily nest survival, breeding phenology, patterns of food availability, and degree of matching to lunar phase. Despite this variation, per pair productivity remained seemingly high for a bird species with a recorded lifespan >15 years (Cink et al., 2017). However, patterns in daily nest survival suggest that food abundance, availability of moonlight, and nighttime temperature might all influence productivity. Therefore, given that whip‐poor‐wills and other nightjars are showing some of the steepest population declines within the rapidly declining aerial insectivore guild (Blancher et al., 2007), we recommend that complex patterns of resource phenology should be explored further, including how juvenile survival and recruitment may differ between matched and mismatched broods. At the very least, seasonal changes in prey abundance and accessibility should be considered when assessing conservation threats for this unique group of lunarphilic nocturnal insectivores.

## CONFLICT OF INTEREST

None declared.

## AUTHOR CONTRIBUTIONS

PE conceived the idea; PE and JN coordinated data collection; PE and DG conducted statistical analyses; PE wrote manuscript. All authors contributed to designing methodology, interpretation of results, manuscript revision, and gave final approval for publication.
